# MiR-150-5p May Contribute to Pathogenesis of Human Leiomyoma via Regulation of the Akt/p27^Kip1^ Pathway In Vitro

**DOI:** 10.3390/ijms20112684

**Published:** 2019-05-31

**Authors:** Jae Hoon Lee, Young Sik Choi, Ji Hyun Park, Heeyon Kim, Inha Lee, Young Bin Won, Bo Hyon Yun, Joo Hyun Park, Seok Kyo Seo, Byung Seok Lee, SiHyun Cho

**Affiliations:** 1 Department of Obstetrics and Gynecology, Severance Hospital, Yonsei University College of Medicine, Seoul 03722, Korea; jhlee126@yuhs.ac (J.H.L.); YSCHOI08@yuhs.ac (Y.S.C.); IHLEE86@yuhs.ac (I.L.); YOUNGBINW@yuhs.ac (Y.B.W.); GARFIELDZZ@yuhs.ac (B.H.Y.); tudeolseo@yuhs.ac (S.K.S.); DR222@yuhs.ac (B.S.L.); 2 Institute of Women’s Life Medical Science, Yonsei University College of Medicine, Seoul 03722, Korea; KIMHY@yuhs.ac (H.K.); BEANPEARL@yuhs.ac (J.H.P.); 3 Department of Obstetrics and Gynecology, Gangnam Severance Hospital, Yonsei University College of Medicine, Seoul 06273, Korea; JIHYUNPARK906@yuhs.ac

**Keywords:** leiomyoma, microRNA 150-5p, Akt, p27^Kip1^

## Abstract

Uterine leiomyoma is found in ~50–80% of women of a reproductive age and is the most common reason for hysterectomy. Recently, posttranscriptional gene silencing by microRNAs (miRs) has been reported as a mechanism for regulating gene expression stability in the pathogenesis of uterine leiomyomas. In this study, miR microarray analysis of leiomyomas and paired myometrial tissue revealed numerous aberrantly expressed miRs, including miR-150. In functional assays, transfection with miR-150 mimic resulted in decreased migration and fibrosis, implying an inhibition of leiomyoma growth. To identify the target genes of miR-150 in leiomyoma, gene set analysis and network analysis were performed. To overcome the limitations of in silico analysis, changes in expression levels of hallmark genes in leiomyoma after transfection with a miR-150 mimic were also evaluated using qRT-PCR. As a result, the Akt/p27^Kip1^ pathway was presumed to be one of the target pathways of miR-150. After transfecting cultured leiomyoma cells with the miR-150 mimic, expression levels of its target gene Akt decreased, whereas those of p27^Kip1^ increased significantly. Our results suggest that miR-150 affects the cell cycle regulation in uterine leiomyoma through the Akt/p27^Kip1^ pathway.

## 1. Introduction

Uterine leiomyoma, the most common reason for hysterectomy, is diagnosed in ~50–80% of women of a reproductive age, resulting in high sociomedical expenses [1,2,3,4]. It is clear that ovarian steroids are essential for the pathophysiology of leiomyoma growth, and therefore the use of drugs targeting ovarian steroids has served as the mainstream treatment strategy [3]. However, symptomatic uterine leiomyomas require continuous treatment until menopause, and currently available drugs are difficult to use for long periods. Gonadotropin-releasing hormone agonists cause significant bone loss after 6 months of therapy [5]. Ulipristal acetate, a selective progesterone receptor modulator that has been in the spotlight for treatment of uterine leiomyoma in the past few years, has recently been reported to cause hepatotoxicity resulting in four cases of liver transplantation, and its safety therefore needs to be verified [6].

For the past decade, growing evidence indicates that local expression of many autocrine/paracrine mediators serve as key regulators of cell-cycle progression, cellular hypertrophy, extracellular matrix accumulation, and apoptosis in the pathophysiology of uterine leiomyoma. In particular, posttranscriptional gene silencing by microRNAs (miRs) has been reported to regulate gene expression stability in the pathogenesis of uterine leiomyomas [7]. Previous studies have identified the expression profile of a large number of miRs in leiomyoma and provide support for altered expression and regulatory function of let 7, miR-21, miR-29, miR-200, and the miR-25-93-106 cluster in leiomyoma and matched myometrium [8,9,10,11,12]. Correlation between miRs and leiomyoma remains poorly understood, and identifying more links between components of the complex network in leiomyoma formation and growth may provide information to establish future therapeutic options for the disease. A recent study has shown that miR-122-targeted therapies have improved treatment outcomes in patients with hepatitis C [13].

Thus, we aimed to investigate aberrantly regulated miRs in leiomyomas using microarrays and quantitative real-time polymerase chain reaction (RT-PCR). We then identified the target genes of these miRs and tested the effects of transfecting miR-150-5p into cultured leiomyoma cells to determine whether it might function as a tumor suppressor in vitro.

## 2. Results

### 2.1. Clinical Characteristics and miR Profiles of Uterine Leiomyoma

Table 1 summarizes the clinical characteristics of 13 participants of this study. The median age of the participants was 44 and the median size of the uterine leiomyoma was 7.4 cm in its largest diameter. 

miR microarray analysis of leiomyomas and paired myometrial tissues revealed that numerous miRs were aberrantly expressed in uterine leiomyoma, and the degree of abnormal miR expression in uterine leiomyomas was evaluated using fold change (FC). With 1.5 FC as a cut-off value, 250 miRs showed aberrant expression among the 6,658 human miRs, whereas with 2.0 FC 124 miRs showed differential expression. Finally, six miRs, three of which were upregulated (hsa-miR-483-5p, hsa-miR-378d, and miR-196b-3p) and three of which downregulated (miR-1 50-5p, miR-139-5p, and miR-140-3p), were found to have differential expression with a statistically significant fold change (Table 2). After a database search and literature review, miR-150 was selected for further validation.

**Figure 1:**
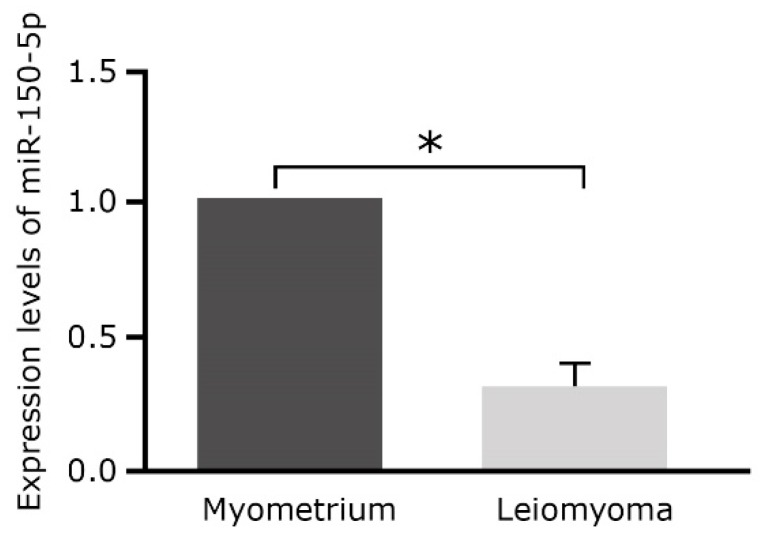
Relative expression of miR-150 was significantly decreased in uterine leiomyomas (*n* = 13) compared to matched myometrium (*n* = 13) according to qRT-PCR analysis (*p* < 0.01). miR-150, microRNA-150-5p; qRT-PCR, quantitative real-time polymerase chain reaction. (* *p* < 0.01).

To confirm the miR microarray results, relative expression of miR-150 in uterine leiomyomas and matched myometrium was validated using qRT-PCR (Figure 1), which revealed that miR-150 expression levels were reduced 0.33 times in leiomyoma compared to myometrium (*p* < 0.01).

### 2.2. Effects of the miR-150 Mimic on Cell Migration and Collagen Gel Contraction

**Figure 2:**
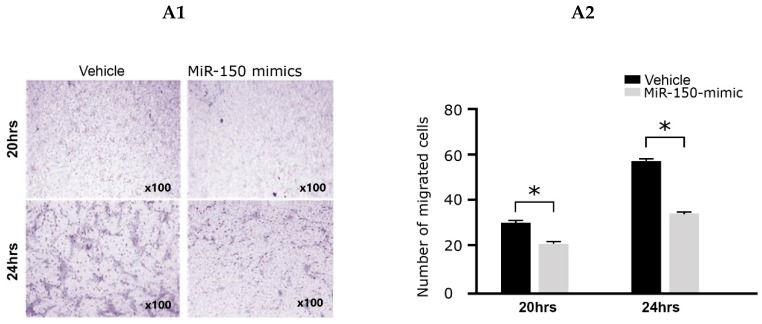

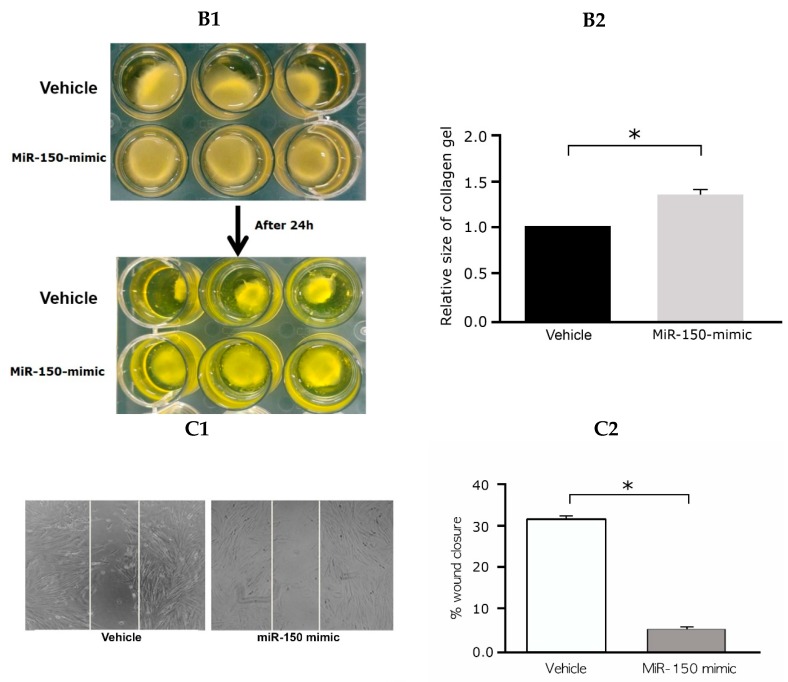
Effects of miR-150 on cell migration and fibrosis of cultured leiomyoma cells. (**A1**,**A2**) miR-150 transfection decreased the migration of cultured leiomyoma cells. Migration assays showed a significant decrease in cell numbers at 20 h (cell count: 28 vs. 20, *p* < 0.05, *n* = 4) and 26 h (cell count: 56 vs. 34. *p* < 0.05, *n* = 4) after miR-150 mimic transfection compared with vehicle transfection. (**B1**,**B2**) miR-150 decreased fibrosis in cultured leiomyoma cells. Collagen gel contractility of leiomyoma cells was evaluated using the collagen gel contraction assay. After 24 h of miR-150 mimic transfection, collagen gel contraction was significantly less than that of the control group (relative contraction gel diameter at 24 h after transfection: 1 vs. 1.36, *p* < 0.05, *n* = 4). (**C1**,**C2**) Wound healing assay, the percentage of wound closure was significantly reduced in miR-150-transfected leiomyoma cells (31.76 ± 0.84 vs. 4.65 ± 2.36, *p* = 0.001, *n* = 4). miR-150, microRNA-150-5p. (* *p* < 0.05).

To determine the effects of miR-150 on cell migration, cultured leiomyoma cells were transfected with the miR-150 mimic. Migration assays showed a significant decrease in cell number at 20 h (cell count: 28 vs. 20, *p* < 0.05, *n* = 4) and 24 h (cell count: 56 vs. 34. *p* < 0.05, *n* = 4) after miR-150 mimic transfection compared with that in miR-negative cultured leiomyoma cells, 48 h after miR-150 transfection (Figure 2(A1,A2)). Collagen gel contractility of leiomyoma cells was evaluated using the collagen gel contraction assay. After 24 h of miR-150 mimic transfection, collagen gel contraction was significantly reduced compared with that of the control group (relative contraction gel diameter at 24 h after transfection: 1 vs. 1.36, *p* < 0.05, *n* = 4) (Figure 2(B1,B2)). In the wound-healing assay, the percentage of wound closure was significantly reduced in miR-150-transfected leiomyoma cells (31.76 ± 0.84 vs. 4.65 ± 2.36, *p* = 0.001, *n* = 4) (Figure 2(C1,C2)).

### 2.3. Gene Set Analysis and Network Analysis of miR-150 Predicted Target Genes and Leiomyoma Related Genes

**Figure 3:**
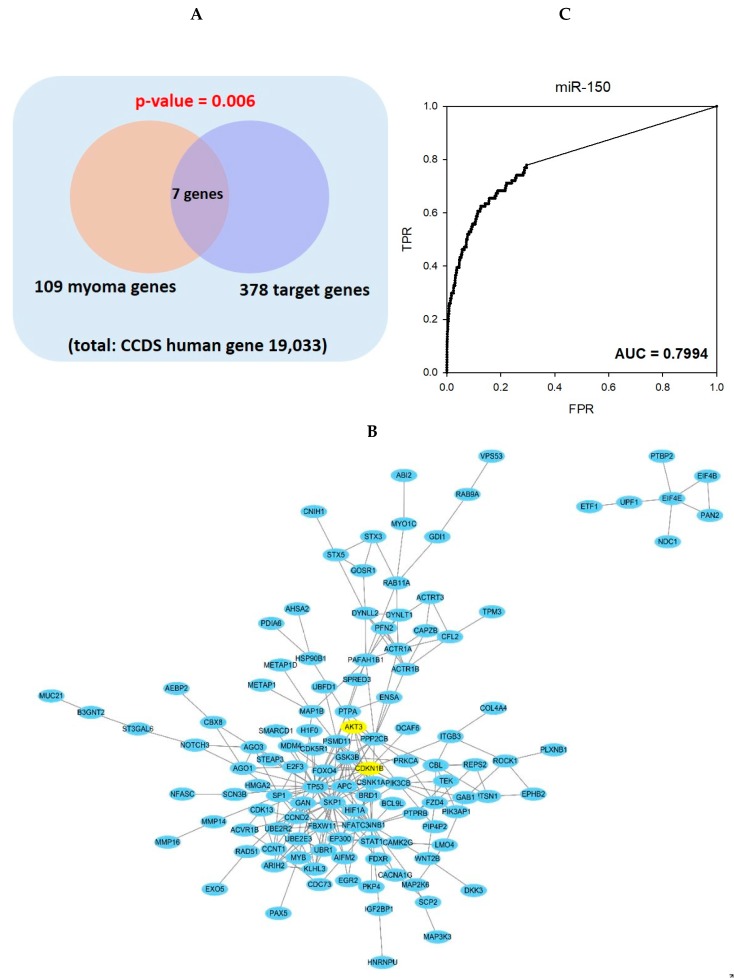
(**A**) Gene set analysis: predicted target genes of miR-150 contain 7 of the 109 previously known leiomyoma related genes. (**B**) In the STRING network model, the predicted target genes of miR-150 are forming modules of sufficient size. Akt and p27^Kip1^, the predicted target genes for miR-150 in this study, was shown in yellow. (**C**) In gene prioritization using the STRING network, the target genes of miR-150 were able to predict leiomyoma genes sufficiently and showed a high AUC prediction power (AUC-0.7994).

To find predicted target genes of miR-150 in leiomyoma, we performed a hypergeometric test of 378 miR-150 target genes predicted from targetscan (http://www.targetscan.org) and 109 known leioyoma-related genes from previously published papers [3,14,15,16,17,18]. As a result, a total of 7 genes-TP53, CTNNB1, HMGA2, PIK3CB, CCND2, GSK3B, and p27^Kip1^ (CDKN1B), were common to both groups and showed a statistical significance of *p*-value 0.006 (Figure 3A).

Using the high confident link of STRING 10.5, the predicted target genes of miR-150 constituted a relatively large-scale subnetwork (Figure 3B). In gene prioritization using the STRING network, the target genes of miR-150 were able to predict leiomyoma genes sufficiently and showed a high AUC prediction power (AUC-0.7994) (Figure 3C). Among the predicted target genes, p27^Kip1^ (CDKN1B) was selected for further validation.

### 2.4. p27^Kip1^ mRNA Expression Levels Decrease in Leiomyoma 

**Figure 4:**
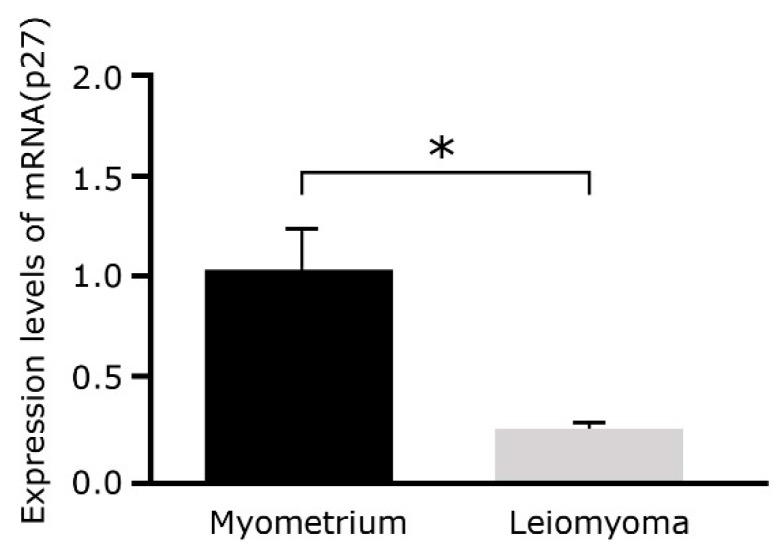
Expression of p27^Kip1^ mRNA was decreased 0.23 times in leiomyoma tissues (*n* = 7) compared to myometrial tissue (*n* = 7) according to qRT-PCR analysis (*p* < 0.01). qRT-PCR, quantitative real-time polymerase chain reaction. (* *p* < 0.01)

To confirm the network analysis results, relative expression of p27^Kip1^ (CDKN1B), a predicted target gene of miR-150, in uterine leiomyomas and matched myometrium was assessed using qRT-PCR (Figure 4). p27^Kip1^ mRNA expression levels decreased 0.23 times in leiomyoma tissues compared to myometrial tissue (*p* < 0.01).

### 2.5. Effects of a miR-150 Mimic on the Expression of Markers of Cell Cycle, Invasion, Apoptosis, and Fibrosis in Cultured Leiomyoma Cells

**Figure 5:**
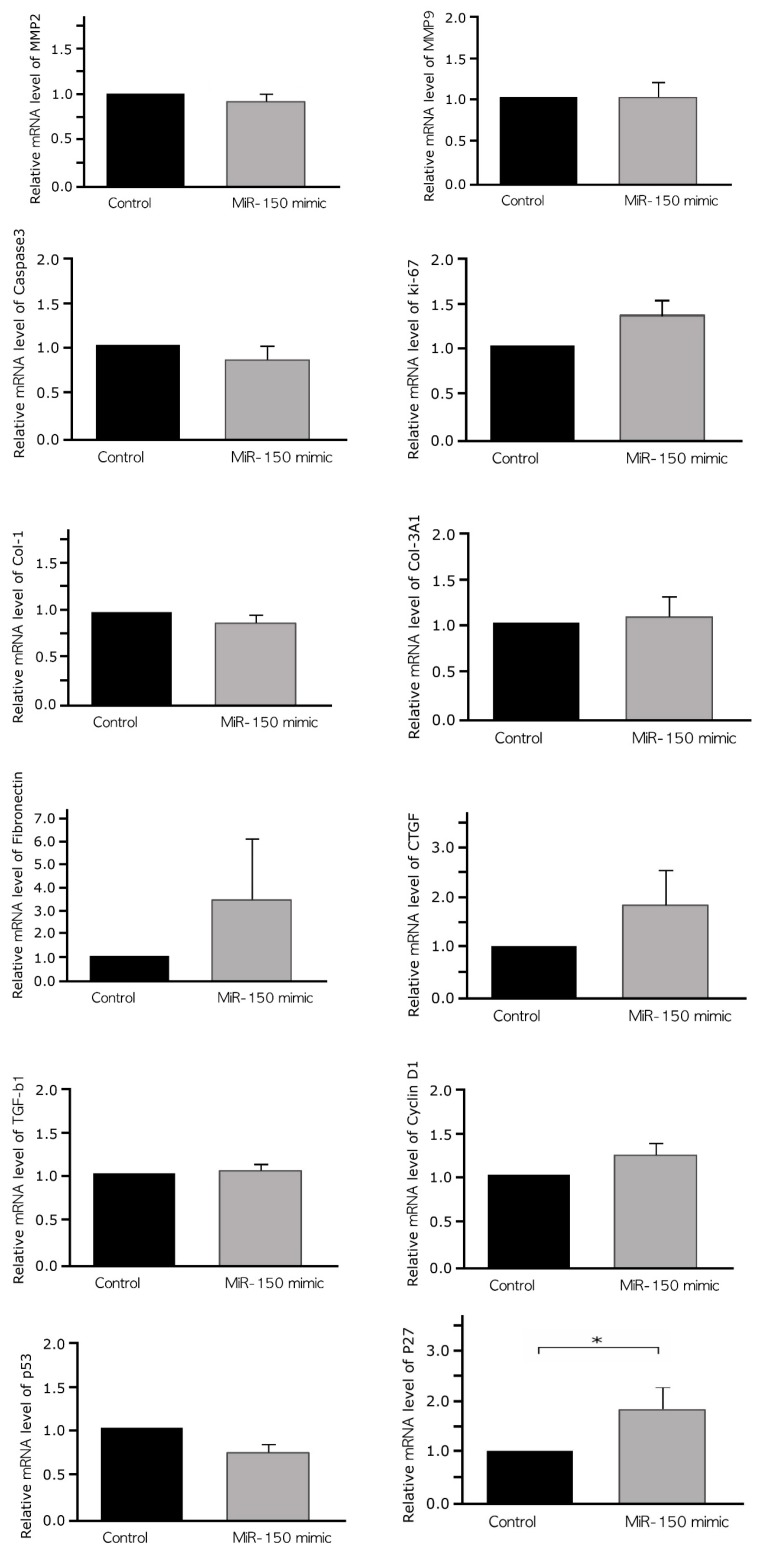
qRT-PCR analysis of mRNA levels of markers of invasion (MMP2 and MMP9), apoptosis (caspase 3), cell proliferation (Ki67), fibrosis (Col-1, Col3A-1, fibronectin, CTGF, and TGFβ-1), and cell cycle (cyclin D1 and p53) 48 h after transfection with the miR-150 mimic. mRNA levels of each sample were normalized to that of GAPDH expression (*n* = 7). qRT-PCR, quantitative real-time polymerase chain reaction; MMP2, matrix metalloproteinase-2; MMP9, matrix metalloproteinase-9; Col-1, collagen type 1; Col-3A1, collagen type 3 α1; CTGF, connective tissue growth factor; TGF-β1, transforming growth factor β1; p27^Kip1^, cyclin-dependent kinase inhibitor 1B; CCND1, cyclin D1; miR-150, microRNA-150-5p. (* *p* < 0.05).

By in silico analysis, we identified predicted target genes of miR-150 in leiomyoma. However, miRs act tissue specifically, whaereas STRING and Targetscan are neither tissue-based database nor disease-based database. Therefore, there may be a difference from the actual. To overcome these limitations, the effects of miR-150 on the expression of markers of apoptosis, invasion, fibrosis, and cell cycle in cultured leiomyoma cells were evaluated using qRT-PCR (Figure 5). Marker selection was determined by reference to review articles [14,17]. To determine transfection efficiency, miR-150 expression was quantified 48 h after transfection of cultured leiomyoma cells with a miR-150 mimic. Treatment with miR-150 resulted in a 100-fold increase in miR-150 expression levels compared with that observed after hsa-miR-negative control treatment.

The levels of matrix metalloproteinase (MMP)-2 and MMP9 (0.85-fold increase in MMP2, *p* = 0.70; 0.95-fold decrease in MMP9, *p* = 0.70), markers of invasion; caspase-3 (0.82-fold increase, *p* = 0.70), a marker of apoptosis; and Ki67 (1.39-fold increase, *p* = 0.70), a marker of cell proliferation; collagen type 1 (Col-1) (0.84-fold increase, *p* = 0.10), collagen type 3 alpha 1 (Col-3A1) (1.08-fold increase, *p* = 1.00), connective tissue growth factor (CTGF) (1.81-fold increase, *p* = 0.333), fibronectin (3.52-fold increase, *p* = 1.00), and transforming growth factor (TGF)-β1 (1.05-fold increase, *p* = 1.00), markers of fibrosis; and p53 (0.73-fold decrease, *p* = 0.100) and cyclin D1 (CCND1) (1.21-fold increase, *p* = 0.209), cell cycle markers were not significantly altered after miR-150 transfection. In contrast, the levels of p27^Kip1^ were significantly increased after miR-150 transfection (1.29-fold increase, *p* = 0.026). 

### 2.6. Effects of the miR-150 Mimic on Akt and p27^Kip1^ Expression in Leiomyoma 

**Figure 6:**
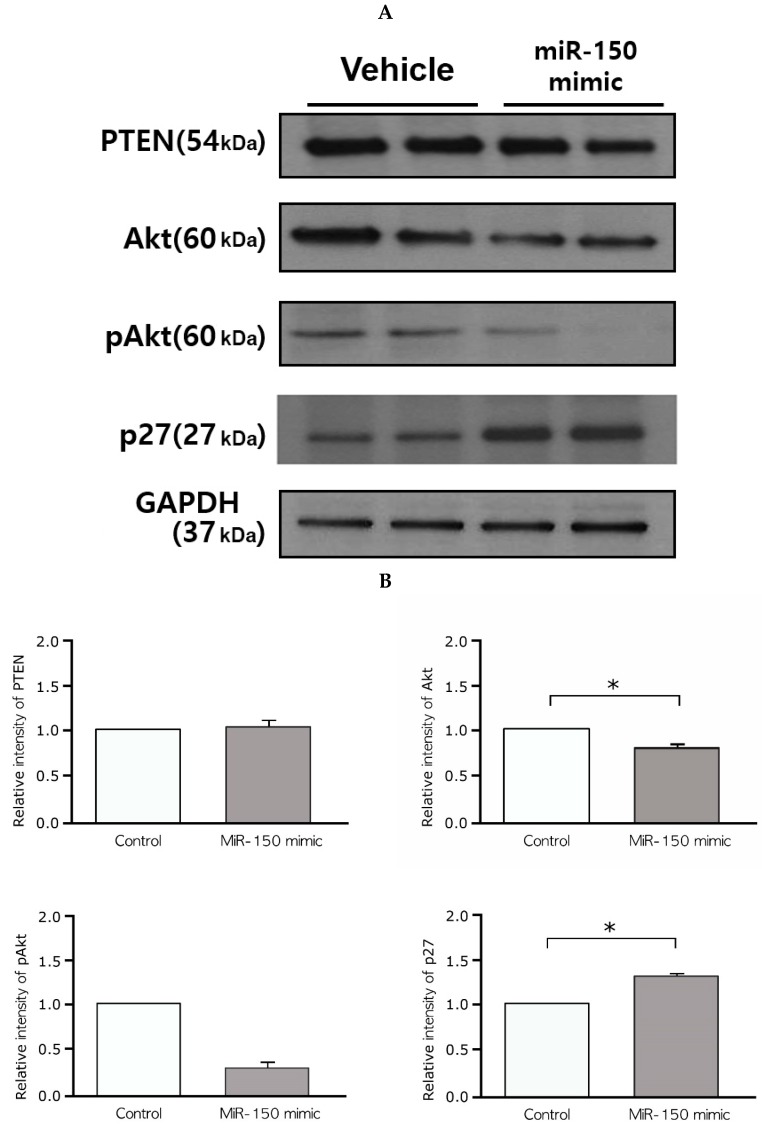
(**A**,**B**) Western blots showing decreased Akt (*p* = 0.028) and pAkt (*p* = 0.068) and increased p27^Kip1^ protein (*p* = 0.043) in cultured leiomyoma cells at 48 h after miR-150 transfection. In contrast, PTEN expression did not change significantly (*p* = 0.180) after miR-150 mimic transfection (*n* = 6). miR-150, microRNA-150-5p; pAkt, phosphorylated Akt; PTEN, phosphatase and tensin homolog. (* *p* < 0.05).

The results of differential expression of hallmark genes after miR-150 mimic transfection and in silico analysis suggested that p27^Kip1^ (or CDKN1B) is one of the target genes of miR-150 in leiomyoma. However, Western blot analysis revealed increased expression of p27^Kip1^ after miR-150 mimic transfection (Figure 6A). Considering the posttranscriptional gene silencing effect of miRs, it was presumed that there is a role for other genes. Additional in silico analysis suggested that Akt (protein kinase B or PKB)–p27^Kip1^ pathway as a target pathway of miR-150 in uterine leiomyoma.

After 48 h of treatment with the miR-150 mimic, we evaluated the expression of Akt, phosphorylated Akt (pAkt), phosphatase and tensin homolog (PTEN), and p27^Kip1^ by Western blot analysis. Decreased levels of Akt (0.670-fold decrease, *p* = 0.028, 60 kDa) and pAkt (0.34-fold decrease, *p* = 0.068, 60 kDa) and increased levels of p27^Kip1^ (1.33-fold increase, *p* = 0.043, 27 kDa) were observed. In contrast, the levels of PTEN, a well-known tumor suppressor that deactivates the phosphatidylinositol 3-kinase (PI3k)-Akt pathway, did not change significantly after miR-150 mimic transfection (0.68-fold decrease, *p* = 0.180, 54 kDa) (Figure 6A,B).

## 3. Discussion

MiRs are non-coding RNAs that regulate biological processes by pairing with the untranslated region (UTR) of target mRNAs to repress their effective translation. MiRs play an important regulatory role in gene expression stability. Moreover, accumulated evidence indicates the potential involvement of genomic instability in leiomyoma formation and growth [7].

Previous studies have found that dysregulated miRs are inversely correlated with their target genes at the protein level [19]. Patterns of inverse association of miRs with mRNA expression in uterine leiomyomas revealed an involvement of multiple candidate pathways, including extensive transcriptional reprogramming, cell proliferation control, decreased programmed cell death, mitogen-activated protein kinase (MAPK), TGF-β, WNT, Janus kinase/signal transducers and activators of transcription signaling, remodeling of cell adhesion, and cell–cell and cell–matrix interactions [19]. In a recent study, miR-29c, miR-200c, and miR-93 3 were reported to regulate cell proliferation of leiomyoma via their effects on key cell cycle regulatory proteins including E2F transcription factor 1(E2F1), Cyclin D1 (CCND1), and CDK2 in vitro [20]. Moreover, in vivo evidence for the tumor suppressor function of miR-29b was recently provided using a kidney capsule transplant model of leiomyoma [21].

In this study, miR-150 was aberrantly expressed in leiomyomas. Because uterine leiomyomas are steroid hormone–sensitive tumors, miRs associated with sex-steroid hormones in breast and prostate cancers have also been investigated in these tumors, and Let-7, miR-21, miR-34a, miR-125b, and miR-150 were identified [14]. In particular, Let-7and miR-21 have been studied extensively. Let-7 is known to have an antiproliferative effect on uterine leiomyomas by repressing its target gene high-mobility group A2 (HMG A2), which is a frequently expressed protein in leiomyomas [22]. Furthermore, Let-7 may also contribute to the malignant transformation of leiomyoma. In a cohort of 35 leiomyosarcoma patients, a significantly decreased Let-7 expression and overexpression of HMGA 2 were observed in leiomyosarcoma tissue. In the same study, growth of leiomyosarcoma cells were repressed when treated with a Let-7 inhibitor [23]. MiR-21 expression is also known to be dysregulated, having an impact on cellular apoptosis and translation in leiomyoma cells [24]. In addition, a recent study reported that miR-21 increases gene and protein expression of TGF-β3 and changes the expression of extracellular matrix genes such as fibronectin, collagen 1A1, CTGF, Versican, and DPT in vitro [25]. Interestingly, the dysregulation of miR-21 is known to be involved not only in leiomyoma but also in various OB/Gyn diseases such as endometrial cancer, endometriosis, preeclampsia, and fetal growth restriction through different mechanisms [26,27,28].

On the other hand, how miR-150 specifically affects tumorigenesis of this disease remains unclear even after more than a decade since the association of miR-150 with leiomyoma was reported. [29]. The role of miR-150 is relatively well known in hematologic malignancies such as malignant lymphoma, in which miR-150 functions as a tumor suppressor by deactivating the PI3K/Akt pathway [30]. Similarly, our results suggest that the inhibitory function of miR-150 on leiomyomas is related to dysregulation of the cell cycle through the Akt/p27^Kip1^ pathway.

The Akt pathway is tightly regulated in a normal cell and plays a central role in modulating cell survival, proliferation, migration, differentiation, and apoptosis [31]. Peng et al. [32] reported that the Akt pathway is activated in approximately 30% of fibroids as determined by immunohistochemistry, and remarkably higher levels of phosphorylated (Ser473)-Akt were observed in leiomyoma tissues than in matched myometrial tissues using Western blot analysis. In fact, the PI3K/Akt-mTOR pathway has been identified as one of the most upregulated signaling pathways in leiomyomas, based on evidence from protein and transcriptional profiling of human leiomyomas, as well as in an Eker rat animal model [33]. Moreover, the Akt inhibitor MK-2206 was found to promote caspase-independent cell death and inhibit leiomyoma growth in a xenograft model, although its clinical use is limited owing to the side effects [34].

Although both Akt and p27^Kip1^ are predicted target genes of miR-150, in silico analysis suggested that p27^Kip1^ plays an important role in leiomyoma pathophysiology. However, Western blotting demonstrated that miR-150 actually acts on Akt/p27^Kip1^ pathway, which shows the difference between in silico analysis and reality. After transfection of miR-150 mimic in cultured leiomyoma cells, the expression levels of Akt and pAkt, known to be elevated in leiomyomas, significantly decreased. However, the expression of p27^Kip1^, known to be downregulated in leiomyomas, significantly increased [31,32,34,35]. Akt can decrease p27^Kip1^ gene expression levels through various mechanisms, such as targeting forkhead transcription factors, loss of PTEN function, impairing nuclear import of p27^Kip1^, and phosphorylation of p27^Kip1^ [33,36,37].

Cell proliferation is a hallmark event in neoplasia, and a large proportion of abnormal cell growth is cell cycle-dependent. In normal tissues, cell cycle kinase inhibitors such as p27^Kip1^ suppress inappropriate responses to tumorigenic stimuli. p27^Kip1^ is known to block the progression of cells from G1 to S phase, and has been demonstrated to control growth and cell cycle progression in human uterine leiomyoma as well as in certain kinds of cancers [38,39]. In vivo, upregulation of p27^Kip1^ by flavopiridol, an anticancer drug, strongly inhibits the growth of uterine leiomyoma cells in xenografted tumors and its effects correlate with the upregulation of p27^Kip1^ [40]. In this study, although miR-150 may target multiple pathways and Akt has various downstream effects, cell cycle arrest due to overexpression of p27^Kip1^ is likely to contribute to decreased of migration, fibrosis, and wound healing of cultured leiomyoma cells. Results that markers involved in extracellular matrix proliferation have not changed after miR-150 transfection support this indirectly.

PTEN is a well-known tumor suppressor that antagonizes PI3K by converting PI(3,4,5)P3 into PI(4,5)P2. Loss of PTEN function leads to over-activation of the PI3/Akt pathway, which is common in cancer cells [41]. Although PTEN is not a predicted target gene of miR-150, we evaluated the expression of PTEN in miR-150-transfected leiomyoma cells to determine whether the effects of miR-150 on leiomyoma reflect higher levels of Akt/p27^Kip1^ pathway activation.

As mentioned above, not only Akt but also p27^Kip1^ are known miR-150 target genes. Although miR-150 repressed Akt rather than p27^Kip1^ in our results, miR-150 inhibits p27^Kip1^ directly by binding to the 3′ UTR of p27^Kip1^ mRNA in other diseases such as prostate cancer [42], which shows the tissue-specific nature of miR expression. Moreover, miR-150 is an oncogene in several types of cancers, including breast, gastric, and lung cancers, and upregulated miR-150 has been reported to be a poor prognostic factor in these diseases [42,43,44,45,46]. However, several previous studies have reported that miR-150 is downregulated in leiomyomas as well as in several hematologic malignancies such as mantle cell, cutaneous T-cell, and Burkitt lymphomas [47,48,49]. The present study also demonstrated that miR-150 transfection effectively reduced the migration potential of leiomyoma cells in vitro, which suggests that miR-150 may inhibit tumor growth of cultured leiomyoma cells. 

This study has several limitations. First, the results were based on an in vitro evaluation. To elucidate the role of miR-150 in leiomyoma, in vivo studies are needed. Second, although there are previous reports that p27^Kip1^ is decreased in leiomyoma compared to matched myometrium [50,51], in order to draw a precise conclusion, reconfirming the baseline expression level of p27^Kip1^ in leiomyoma using same samples which were used assessing changes of Akt and p27^Kip1^ after miR-150 transfection is necessary.

In conclusion, miR-150 is aberrantly expressed in leiomyoma compared to its paired myometrium, and miR-150 transfection decreased Akt and increased p27^Kip1^ expression levels. Moreover, cultured leiomyoma cells transfected with miR-150 showed significantly decreased fibrosis and cell migration capacity in vitro. The present study does not address the mechanism underlying the loss of miR-150 expression in leiomyoma. As shown in Figure 3B, there are several pathways associated with miR-150 in leiomyoma, and further study is needed regarding the role of other pathways other than the Akt/p27^Kip1^ pathway in the pathophysiology of leiomyoma. It is also unclear whether miR-150 reduction is the primary cause of uterine leiomyoma or an intermediate phase of leiomyoma pathogenesis. However, our results suggest that miR-150 affects the cell cycle regulation in uterine leiomyoma through the Akt/p27^Kip1^ pathway. Although the pathogenesis of leiomyoma remains unclear, this study provides a basis for investigating the underlying mechanisms responsible for human uterine leiomyoma.

## 4. Materials and Methods 

### 4.1. Study Subjects and Tissue Specimens

Thirteen women participated in this study after providing written informed consent. Uterine leiomyoma and adjacent myometrial (within 2 cm) tissues were collected from hysterectomy specimens obtained from patients with symptomatic disease between June 2015 and July 2016. All tissue samples were collected within 1 h of surgery, and tissues were stored at −80 °C before being used to determine microRNA, mRNA, and protein levels by microarray, RT-PCR, and Western blotting analyses, respectively. The tissues were rinsed in cold phosphate-buffered saline three times and then cut into 4-mm^3^ pieces and placed in vials containing RNALater (Ambion, Austin, TX, USA) for nucleic acid preservation. Tissue vials were kept at 4 °C for 24 h to allow penetration of RNALater. Vials were then stored at −80 °C until RNA isolation. Table S1 describes where 13 samples were used in each experiment. Study participants were not under any medication such as hormonal therapy for 3 months prior to surgery based on the last menstrual period and endometrial histology. The study protocol was approved by the institutional review board (3-2015-0249, approval date 26 August 2015) of Gangnam Severance Hospital (Seoul, Korea).

### 4.2. Cell Culture of Leiomyoma and Myometrial Smooth Muscle Cells

Leiomyoma tissue samples obtained from women with uterine leiomyoma during hysterectomy were cut into small pieces of approximately 2~5 mm^3^ in size and incubated in Dulbecco’s modified Eagle’s medium without phenol red (Sigma–Aldrich, St Louis, MO, USA) containing collagenase type I 2.0 mg/mL (Gibco, Waltham, MA, USA) and 1% antibiotic-antimycotic mixture containing 100 IU/mL penicillin, 100 mg/mL streptomycin, and 10% heat-inactivated fetal bovine serum (FBS) for 45 min at 37 °C in a shaker. The digested tissue was subsequently cultured using the explant method in a humidified incubator at 37 °C and 5% CO_2_ for 2–4 h; cell passages were routinely conducted using Versene solution-EDTA (Gibco). Of the samples obtained from 13 patients, primary single cell lines from 9 leiomyomas were used for cell culture, because only nine leiomyoma tissue samples had a sufficient number of cells for Western blotting and functional assays. Cultured cells from passage numbers 2 to 4 were used in the experiments.

### 4.3. miR Microarray Analysis

For quality control, the RNA purity and integrity were evaluated using an ND-2000 spectrophotometer (NanoDrop, Wilmington, OH, USA) and an Agilent 2100 Bioanalyzer (Agilent Technologies, Palo Alto, CA, USA). The Affymetrix Genechip miRNA 4.0 array (Affymetrix, Santa Clara, CA, USA) was used according to the manufacturer’s instructions. RNA samples (1000 ng) were labeled with the FlashTag Biotin RNA Labeling Kit (Genisphere, Hatfield, PA, USA). Labeled RNA was quantified, fractionated, and hybridized to the miRNA microarray following the manufacturer’s instructions. Labeled RNA was heated to 99 °C for 5 min and then to 45 °C for 5 min. RNA-array hybridization was performed with agitation at 60 rotations/min for 16 h at 48 °C on an Affymetrix 450 Fluidics Station. Chips were washed and stained using a Genechip Fluidics Station 450 (Affymetrix). Next, chips were scanned using an Affymetrix GCS 3000 scanner (Affymetrix). Signal values were computed using the Affymetrix GeneChip Command Console software.

Raw data were extracted automatically as per the Affymetrix data extraction protocol using the Affymetrix GeneChip Command Console Software. CEL file import, miRNA level RMA + DABG, all analysis, and result export were performed using the Affymetrix Expression Console Software. Array data were filtered with probes annotated by species. Comparative analysis between test and control samples was carried out using fold-change and paired T-test, in which the null hypothesis was that no difference existed among two groups. The false discovery rate (FDR) was controlled by adjusting the p value using the Benjamini-Hochberg algorithm. For the DEmiRNA set, hierarchical cluster analysis was performed using complete linkage and the Euclidean distance as a measure of similarity. Statistical tests and visualization of differentially expressed genes were conducted using the R statistical language v. 3.1.2. software.

Intensity data file import, miRNA level robust multichip average detection above background-All analysis, and result export were performed using the Affymetrix Expression Console Software (version 1.4.1.46). Array data were filtered using probes annotated by species. Comparative analysis between test samples and control samples was performed using a fold-change approach. Visualization of differentially expressed genes and statistical tests were performed using the R statistical language v. 2.15.0 software.

### 4.4. RNA Isolation and Quantitative Real-Time PCR

To assess miR expression levels, RNA was extracted from leiomyoma and matched myometrial tissues using the miRVana RNA Isolation Kit (Ambion) as per the manufacturer’s instructions; 30 μL of nuclease-free water was used to elute RNA. A Nanodrop ND-2000 spectrophotometer was used to determine the RNA yield. A total of 10 ng of isolated RNA and the Taqman MicroRNA Reverse Transcription Kit (Applied Biosystems, Thermo Fisher Scientific, Baltics, Lithuania) were also used. qRT-PCR for miRs was performed using a Taqman Universal Master Mix II, with uracil-N-glycosylase (UNG) (Applied Biosystems), with sets for miR-150 and U6 small nuclear RNA (U6 snRNA) (Applied Biosystems). All real-time PCR reactions were performed in triplicate using a 7300 Real Time PCR system; 40 amplification cycles were performed. Relative expression was calculated using the comparative threshold cycle (*C*t) method, and miR levels were normalized to U6 levels [52].

To measure the mRNA levels of MMP2, MMP9, caspase-3, Ki67, Col-1, Col-3A1, CTGF, fibronectin, TGF-β1, p53, cyclin D1, Akt, pAkt, PTEN, and p27^Kip1^, total RNA was isolated from cultured leiomyoma cells using the RNeasy Mini Kit (Qiagen, Hilden, Germany). RNA concentrations were determined using a Nanodrop ND-2000 spectrophotometer (Thermo Fisher Scientific, Waltham, MA, USA). Using 1000 ng of total RNA, cDNA was synthesized with oligo-dT (Superscript III kit, Invitrogen) in a C1000 Thermal Cycler (Bio-Rad, Hercules, California, CA, USA). The resultant cDNA mixtures were stored at −20 °C. Using 2 µL of synthesized cDNA template, qRT-PCR amplification was performed using a 7300 Real Time PCR System (Applied Biosystems). The Power SYBR Green PCR master mix (Applied Biosystems) was used for nucleic acid quantitation in RT PCR. The reaction mixture included the cDNA template, forward and reverse primers, ribonuclease-free water, and the SYBR Green master mix at a 20-µL final reaction volume. Thermal cycling conditions were 95 °C for 5 min, followed by 40 cycles of 95 °C for 30 s, 60 °C for 30 s, and 72 °C for 1 min, and a final extension at 72 °C for 5 min. The Ct and melting curves were calculated using the 7300 software (Applied Biosystems), and each reaction was performed in triplicate. mRNA levels for each sample was normalized to the glyceraldehyde 3-phosphate dehydrogenase (GAPDH) levels.

The primers for MMP2, MMP9, caspase-3, Ki67, Col-1, Col-3A1, CTGF, fibronectin, TGF-β1, p53, cyclin D1, Akt, pAkt, PTEN, PI3K, p27^Kip1^, and GAPDH were as follows: MMP2 forward, 5′-ACC GCG ACA AGA AGT ATG GC-3′ and reverse, 5′-CCA CTT GCG GTC ATC ATC GT-3′; MMP9 forward, 5′-CGA TGA CGA GTT GTG GTC CC-3′ and reverse, 5′-TCG TAG TTG GCC GTG GTA CT-3′; caspase 3 forward, 5′-GGA AGC GAA TCA ATG GAC TCT GG-3′ and reverse, 5′-GCA TCG ACA TCT GTA CCA GAC C-3′; Ki67 forward, 5′-GAA AGA GTG GCA ACC TGC CTT C-3′ and reverse, 5′-GCA CCA AGT TTT ACT ACA TCT GCC-3′; Col-1 forward, 5′-GAG AGC ATG ACC GAT GGA TT-3′ and reverse, 5′-CCT TCT TGA GGT TGC CAG TC-3′; Col-3A1 forward, 5′- TTG TTC ATT CTT GCC GTG TTT C -3′ and reverse, 5′-TCC TCC TAG GGC GTC CTG TT -3′; CTGF forward, 5′-CAT TAA GAA GGG CAA AAA GTG C-3′ and reverse, 5′-CAC ACC CCA CAG AAC TTA GCC-3′; fibronectin forward, 5′-CCA TCG CAA ACC GCT GCC AT-3′ and reverse, 5′-AAC ACT TCT CAG CTA TGG GCT T-3′; TGF-β1 forward, 5′-TGG AAA CCC ACA ACG AAA TC-3′ and reverse, 5′-GGG TTC AGG TAC CGC TTC TC-3′; p53 forward, 5′-GCC CAA CAA CAC CAG CTC CT-3′ and reverse, 5′-CCT GGG CAT CCT TGA GTT CC-3′; cyclin D1 forward, 5′-TGC ATG TTC GTG GCC TCT AA-3′ and reverse, 5′-TCG GTG TAG ATG CAC AGC TT-3′; Akt forward, 5′-TGA AAA CCT TCT GTG GGA CC-3′ and reverse, 5′-TGG TCC TGG TTG TAG AAG GG-3′; PTEN forward, 5′-ATA CCA GGA CCA GAG GAA ACC-3′ and reverse, 5′-TTG TCA TTA TCC GCA CGC TC-3′; p27^Kip1^ forward, 5′-GCA CAC TTG TAG GAT AAG TGA AAT GG-3′ and reverse, 5′-CCT ATT CTA CCC AAC ACA GCA TTT AC-3′; and p53 forward, 5′-GCC CAA CAA CAC CAG CTC CT-3′ and reverse, 5′-CCT GGG CAT CCT TGA GTT CC-3′.

### 4.5. Target Gene Prediction for miR-150

To predict the target genes of miR-150, the effects of miR-150 on hallmark genes of the cell cycle, invasion, apoptosis, and fibrosis were evaluated in cultured leiomyoma cells using qRT-PCR. Next, in silico analysis was conducted. First, gene set analysis of 378 miR-150 target genes predicted from targetscan (http://www.targetscan.org) and 109 known leioyoma-related genes from previously published papers. Second, using the high confident link of STRING 10.5, network-based gene prioritization was performed. Input data were as follows: [Input 1] Network: STRING 10.5 network; [Input 2] Seed: predicted target gene list of miR-150; [Input 3] Validation Set: previously known leiomyoma genes. In prioritizing genes, Naïve Bayes method [53] was used for network propagation. The AUC score was used to calculate the rank of the leiomyoma-related genes.

### 4.6. Protein Isolation and Western Blotting Analysis

Protein extracts were prepared using RIPA lysis buffer (Thermo Fisher Scientific, Rockford, Illinois, IL, USA) containing protease and phosphatase inhibitor cocktail (Thermo Fisher Scientific). Concentrations of total cell lysates were measured using a bicinchoninic acid protein assay kit (Thermo Fisher Scientific). A total of 20 μg of total protein was mixed with 5× sample buffer and heated at 95 °C for 5 min. Samples were loaded onto 10% sodium dodecyl sulfate polyacrylamide gels. After electrophoresis, they were electrotransferred to a polyvinylidene fluoride membrane (Millipore, Billerica, MA, USA) using a Transblot apparatus (Bio-Rad). Membranes were blocked using 5% nonfat skim milk in Tris-buffered saline solution (10 mmol/L Tris-HCl (pH 7.4) and 0.5 mol/L NaCl) with Tween-20 (0.1% vol/vol). Blots were probed using the following primary antibodies: anti-Akt (1:1000; Cell Signaling Technology, Danvers, MA, USA), anti-pAkt (1:1000; Cell Signaling Technology, Danvers, MA, USA), anti-p27^Kip1^ (1:1000; Cell Signaling Technology, Danvers, MA, USA), anti-PTEN (1:1000; Cell Signaling Technology, Danvers, Massachusetts) and anti-GAPDH (1:2000, Abcam, Cambridge, UK); the secondary antibody used was the horseradish peroxidase–conjugated secondary anti-mouse or anti-rabbit antibody (1:2000; Thermo Scientific). Proteins were detected using enhanced chemiluminescence (Santa Cruz Biotechnology, Dallas, TX, USA). Experiments were performed in triplicate.

### 4.7. MiR Transfection

Cells were cultured to 70% to 80% confluence after being seeded into six-well plates and then transfected with hsa-miR-negative as a control or with hsa-miR-150-5p (Thermo Fisher Scientific, Waltham, MA, USA), a chemically synthesized double-stranded RNA that mimics mature endogenous miR. Lipofectamine 2000 (Invitrogen, Carlsbad, CA, USA) transfection reagent was used according to the manufacturer’s instructions to obtain a final concentration of 50 nM. Transfected cells were harvested 48 h after the reagent was added.

### 4.8. Migration Assay

Migration assays for transfected cultured cells were carried out using 8-mm pore size polycarbonate membranes (Millipore, Billerica, MA, USA) and 24-well plates. Freshly trypsinized and washed cells were suspended in serum-free medium, and cells (200 μL, 5 × 10^4^ cells/well) were placed in the top chamber of each insert; medium (600 μL) containing 10% FBS was added into the lower chambers. After incubating for 24 h at 37 °C in a 5% CO_2_ humidified incubator, cells were fixed and stained with crystal violet solution. Cells in the inner chamber were removed using a cotton swab, and cells attached to the bottom side of the membrane were counted and imaged under an inverted microscope (Olympus Corp., Shinjuku, Tokyo, Japan) at 200× magnification over 10 random fields in each well [54].

### 4.9. Collagen Gel Contraction Assay

A sterile solution of bovine type I collagen (Cell Biolabs, San Diego, CA, USA) was prepared per the manufacturer’s instructions. Leiomyoma cells transfected with miR-150 were embedded in collagen gel for three-dimensional cell culture. Briefly, leiomyoma cells were suspended in the collagen solution (3.0 × 10^5^ cells/mL). The collagen/cell mixture (0.5 mL/plate) was dispensed into 24-well culture plates (Corning, New York, NY, USA); the mixture was polymerized at 37 °C for 1 h. Immediately after polymerization, 1 mL culture medium was added to each plate. After incubation for 72 h, the collagen gels were photographed and the gel surface area was measured [55,56].

### 4.10. Wound Healing Assay 

Wound healing assays were performed as described previously, with minor modifications [57]. Briefly, leiomyoma cells transfected with miR-150 (5.0 × 10^5^ cells per well) were cultured in 6-well plates until 80% confluent. The medium was replaced with serum-free medium containing the corresponding drugs, and the medium was collected after 12 h of incubation. The confluent monolayer cells were carefully removed using a 200 μL yellow tip and washed twice with PBS. The previous media were added to the corresponding wells. Cells were photographed at low magnification at time intervals of 0, 12, and 24 h. The wounded area was calculated using the following formula: (mean wounded breadth × mean remained breadth)/mean wounded breadth × 100 (%). Experiments were performed independently in triplicate.

### 4.11. Statistical Analysis

Results are presented as mean ± standard error of the mean (SEM). The data were checked to determine whether they met the requirements for a normal distribution using the Kolmogorov-Smirnov test or the Shapiro-Wilk test. Continuous variables were compared using the Student *t* test, Mann-Whitney U test, or Wilcoxon Signed rank test where appropriate. Fisher’s exact test was used for gene set analysis. SPSS v.23.0 and R statistical language v.2.15.0 were used for statistical analyses, and *p* < 0.05 was considered statistically significant.

## Figures and Tables

**Table 1:** Patient characteristics.

Variables	*N* = 13
Age	44 (37–48)
Name of operation	Hysterectomy
Site of leiomyoma	Intramural
Size of leiomyoma	7.4 cm (3.7–12.2)
Follicular phase	10
Proliferative phase	3

Data are expressed as the median.

**Table 2:** Microarray analysis and significant fold changes of miRNAs between leiomyoma tissues and paired myometrium.

Upregulated			Downregulated		
miRNA	FC (SD)	*p* Value	miRNA	FC (SD)	*p* Value
hsa-miR-196b-3p	2.16 (0.39)	0.04	hsa-miR-139-5p	5.06 (2.31)	0.04
hsa-miR-483-5p	3.1 (0.63)	0.04	hsa-miR-140-3p	2.08 (0.94)	0.02
hsa-miR-378d	1.82 (0.52)	0.04	hsa-miR-150-5p	4.79 (2.29)	0.03

miRNA, microRNA; FC, fold change; SD: standard deviation.

## Supplementary Materials

Supplementary materials can be found at https://www.mdpi.com/1422-0067/20/11/2684/s1.

## Abbreviations

FC	Fold change
miR	microRNA
RT-PCR	Real-time polymerase chain reaction
UTR	Untranslated region
