# Supplementary material for: MiR-150-5p May Contribute to Pathogenesis of Human Leiomyoma via Regulation of the Akt/p27Kip1 Pathway In Vitro
Source: Int J Mol Sci. 2019 May 31;20(11):2684. doi: 10.3390/ijms20112684 (PMC6601023; doi:10.3390/ijms20112684)
Supplement: Supplementary file 1 [file ijms-20-02684-s001.pdf]

Sample	Table2	Figure1	Figure2	Figure4	Figure5	Figure 6
#1	O	O	O	O	O	O
#2	O	O	O	O	O	O
#3	O	O				
#4	O	O				
#5	O	O		O	O	O
#6	O	O		O		O
#7	O	O	O	O	O	O
#8	O	O			O	
#9	O	O	O	O	O	O
#10	O	O		O		
#11	O	O				
#12	O	O			O	
#13	O	O				

Supplementary Table 1. Table2, Figure1 and Figure 4 were experiments using leiomyoma tissue and the remainder were experiments using cultured leiomyoma cells. In Table 2 and Figure 1, all 13 samples were used. Samples #1,#2,#7, and #9 provided enough cells; however, in samples #3,#4,#11,#13, cells were not cultured well and did not provide adequate amounts of cells. Samples #1,#2,#7, and #9 are representative samples of this study and were used in all experimental steps. For the experiment, these four samples were used as a basis and additional samples were added as needed for each experiment.
